# An Evaluation of the Effectiveness of the Modalities Used to Deliver Electronic Health Interventions for Chronic Pain: Systematic Review With Network Meta-Analysis

**DOI:** 10.2196/11086

**Published:** 2019-07-17

**Authors:** Brian W Slattery, Stephanie Haugh, Laura O'Connor, Kady Francis, Christopher P Dwyer, Siobhán O'Higgins, Jonathan Egan, Brian E McGuire

**Affiliations:** 1 School of Nursing and Human Sciences Dublin City University Dublin Ireland; 2 Centre for Pain Research School of Psychology National University of Ireland Galway Ireland

**Keywords:** eHealth, mHealth, digital health, Virtual Reality, chronic pain, systematic review, network meta-analysis

## Abstract

**Background:**

Electronic health (eHealth) is the use of information and communication technology in the context of health care and health research. Recently, there has been a rise in the number of eHealth modalities and the frequency with which they are used to deliver technology-assisted self-management interventions for people living with chronic pain. However, there has been little or no research directly comparing these eHealth modalities.

**Objective:**

The aim of this systematic review with a network meta-analysis (NMA) is to compare the effectiveness of eHealth modalities in the context of chronic pain.

**Methods:**

Randomized controlled trials (N>20 per arm) that investigated interventions for adults with chronic pain, delivered via an eHealth modality, were included. Included studies were categorized into their primary node of delivery.

Data were extracted on the primary outcome, pain interference, and secondary outcomes, pain severity, psychological distress, and health-related quality of life. Pairwise meta-analyses were undertaken where possible, and an NMA was conducted to generate indirect comparisons and rankings of modalities for reducing pain interference.

**Results:**

The search returned 18,470 studies with 18,349 being excluded (duplicates=2310; title and abstract=16,039). Of the remaining papers, 30 studies with 5394 randomized participants were included in the review. Rankings tentatively indicated that modern eHealth modalities are the most effective, with a 43% chance that mobile apps delivered the most effective interventions, followed by a 34% chance that interventions delivered via virtual reality were the most effective.

**Conclusions:**

This systematic review with an NMA generated comparisons between eHealth modalities previously not compared to determine which delivered the most effective interventions for the reduction of pain interference in chronic pain patients. There are limitations with this review, in particular, the underrepresented nature of some eHealth modalities included in the analysis. However, in the event that the review is regularly updated, a clear ranking of eHealth modalities for the reduction of pain interference will emerge.

## Introduction

### Electronic Health

As technological advances pervade every aspect of daily life, there has been a corresponding proliferation in the development and implementation of technological interventions for health-related purposes. Electronic health (eHealth), the broad term for information and communication technologies deployed in health settings, is a growing area of interest as the international research community attempts to address issues facing modern health care [1]. Typically, an eHealth *modality* is considered to be some specific form of technology that is applied in the context of health care [2-4]. Examples of eHealth modalities include internet-based (Web-based health interventions [5-9], telephone-supported (interventions with telephone support from health practitioners) [10], interactive voice response (the use of a phone’s touch-tone keypad to provide responses to automated scripts) [11,12], virtual reality (a 3-dimensional computer-generated environment that the individual can explore, interact with, and manipulate) [13,14], videoconferencing (the use of high-quality real-time video and audio connection via online internet networks) [15], and mobile phone apps (mobile-based or mobile-enhanced programs) that deliver health-related services [16,17]. More detailed definitions of the various types of eHealth interventions are available in Multimedia Appendix 1.

The core value proposition for delivering health care via an eHealth modality is that the barriers experienced by traditional in-person treatment methods are reduced or potentially removed [4,18-21]. For instance, a Web-based eHealth intervention may improve accessibility to treatment, reduce the waiting list duration, and can be delivered more cost-effectively than in-person services [22]. For these reasons, eHealth has gained considerable traction for conditions that are long-term and where there is a shift toward self-management [23-26]. In this context, where ongoing disease management is required, eHealth interventions offer a viable and important support option. Many eHealth solutions have been developed for a variety of chronic illnesses, including diabetes [27,28], breast cancer [29], hypertension [30], cardiovascular disease [16], multiple sclerosis [31], headache [8], and chronic pain [12,14,32-38].

### Electronic Health and Chronic Pain

Chronic pain refers to pain that lasts for more than 3 months [39]. Chronic pain encompasses many diverse conditions, is highly prevalent, and is a leading cause of long-term disability [39]. Much eHealth research has been conducted in the area of chronic (noncancer) pain, and eHealth interventions have shown to be efficacious in reducing pain interference [40]. However, despite the increasing variety of eHealth modalities used for chronic pain, studies typically focus on 1 modality, and as a result, direct comparisons of modalities are rare [22]. Identifying the need to investigate the relative strengths and weaknesses of modality types, Heapy et al conducted a systematic review of eHealth self-management interventions for chronic pain, in which three modality types were evaluated, namely, telephone, interactive voice response, and internet. They concluded that each modality was effective in the context of chronic pain, but no conclusive evidence points to one being more superior than the others.

Notably, Heapy et al began the necessary steps toward ascertaining the varying efficacies of each modality as the *contributing factor* to intervention success. However, the authors recognized certain limitations with their review, such as the breadth of their search strategy (ie, limited to three databases) and the low number (ie, 3) of included eHealth modalities. Moreover, the review included a variety of study designs, and although they reported on the between-condition effect sizes when possible, a quantitative comparison (ie, a meta-analysis) was not conducted. Therefore, one of Heapy et al’s indications for future research was to identify the relative efficacy of modality types through direct comparison.

### Why Is It Important to Do This Review?

Although there are a growing number of eHealth interventions for chronic pain, there is a stark lack of research comparing eHealth modalities in this context. Directly comparing eHealth modalities deployed in chronic pain research could potentially yield important insights into which modalities are more efficacious in what context and for what reasons (eg, treatment fidelity, resource availability, issues with target population, typical engagement levels, and cost efficiency). Thus, from the perspectives of patient well-being, health care provision, and optimizing research interventions, there is an impetus to first identify the most effective modalities for chronic pain and to then investigate why they are the most effective.

The aim of this study was to add to the literature that concerns itself with evaluating eHealth modalities in the context of reducing pain interference for chronic pain patients by directly comparing treatment outcomes across studies that have deployed an eHealth modality. Critically, this review conducted a network meta-analysis (NMA) and quantitatively compared and ranked the eHealth modalities used for interventions in chronic pain, which has not been done before. An NMA is an extension of a meta-analysis and enables multiple treatments to be compared using direct and indirect comparisons across trials using a common comparator [41-43].

### Objective

The objective of this study was to conduct a systematic review and an NMA to evaluate and compare the effectiveness of the eHealth modalities used to deliver interventions (other than drugs) for adults living with chronic noncancer pain.

## Methods

### Protocol and Registration

The systematic review and NMA were conducted and reported in accordance with the Preferred Reporting Items for Systematic Reviews and Meta-Analyses (PRISMA) guidelines and the PRISMA Network Meta-Analysis extension statement (see Multimedia Appendix 2) [44]. The protocol for this study is registered with the International Prospective Register of Systematic Reviews database (registration number: CRD42016035595) [45].

### Outcomes

#### Primary Outcome

Similar to previous research [33,34,46,47], and in accordance with outcome measures outlined by the Initiative on Methods, Measurement, and Pain Assessment in Clinical Trials [48], pain interference was the primary outcome variable. Where pain interference was not reported, pain**-**related disability or a reverse-scored measure of physical functioning was extracted.

#### Secondary Outcomes

Secondary outcomes were measures of pain severity, psychological distress (measures of depression were extracted where available; measures of anxiety and reverse-scored measures of mental health were also acceptable), and health-related quality of life (HRQoL).

### Eligibility Criteria

The eligibility requirements for included studies in this review are outlined in Table 1. All studies included in this review were required to be published in peer-reviewed journals and available in English. The criteria were influenced by a Cochrane review of internet-delivered psychological therapies for chronic pain by Eccleston et al [49].

### Classification

Studies were merged to create nodes representing the primary delivery method (eg, internet). A study was not included in the network if both arms were classified as the same modality without an additional comparator.

### Information Sources

A total of 4 databases, Cochrane Central Register of Controlled Trials (CENTRAL; Cochrane Library), Medical Literature Analysis and Retrieval System Online (MEDLINE), Excerpta Medica dataBASE (EMBASE), and PsycINFO, were searched from inception until November 22, 2017. Necessary changes were made to adapt the search terms for different interfaces. The search strategy is detailed in Textbox 1.

The reference lists of relevant systematic reviews and of included studies were screened to identify any relevant studies. The metaRegister of Controlled Trials [50], Clinicaltrials.gov [51], and the World Health Organization’s International Clinical Trials Registry Platform [52] were also searched.

### Study Selection

Members of the research team screened titles and abstracts to search for duplicate and nonrelevant studies; 10% of the papers were assessed in duplicate. In total, 2 review authors (SH and KF) independently screened full-text papers for inclusion. Studies were included if they (1) were randomized controlled trials (RCTs); (2) had N>20 per arm at each time point; (3) had participants with noncancer-related chronic pain; (4) were delivered via eHealth modality; and (5) measured a suitable pain outcome.

**Table 1:** Eligibility criteria (Population, Intervention, Comparison, Outcome, Study Design) included in this review.

Category	Eligibility criteria
Population	Adults with noncancer-related chronic pain
Intervention	Interventions for managing chronic pain delivered via an electronic health (eHealth) modality
Comparison intervention	At least one of the following: an active eHealth intervention; enhanced control; treatment-as-usual; waiting-list control
Outcome measures	Pain interference; pain severity; psychological distress; health-related quality of life
Study design	Randomized controlled trials

**Search terms.**

(Telecommunications)/ OR (telemedicine OR tele-medicine).mp OR (telehealth OR tele-health).mp OR (ehealth OR e-health).mp OR (mobile health OR mhealth OR m-health).mp OR (ICT).mp OR ((inform* OR communicat* OR interact*) adj6 (computer* OR technolog* OR software)).mp OR ((health* OR treat* OR therap* or intervention* OR assist* OR selfmanag* OR self-manag*) adj6 (computer* OR technolog* OR software)).mp OR (internet)/ OR (internet* OR world wide web OR www OR web-based OR email OR e-mail OR online).mp OR (telephone* OR phone* OR mobile* OR cellphone* OR cellular telephone* OR application* OR app* OR text* OR SMS OR smartphone* OR mobile operating system technolog* OR microcomputer* ).mp OR (virtual reality OR augmented reality OR VR OR AR).mp OR (IVR OR interactive voice response OR voice response unit OR VRU OR speech recognition OR voice recognition).mp
  AND
(Pain)/ OR (Pain Measurement)/ OR (Headache disorders)/ OR (Fibromyalgia)/ OR (pain* OR headache* OR migraine* OR fibromyalgia* OR neuralgia*).mp OR (pain intensity OR pain severity OR pain outcome*) OR (self-reported pain)
  AND
“Chronic pain” OR headache*
  AND
(randomized controlled trial OR randomised controlled trial.pt) OR (controlled clinical trial.pt) OR (randomized.ab OR randomised.ab) OR (placebo.ab) OR (clinical trials as topic.sh) OR (randomly.ab) OR (trial.ti) OR (groups.ti)

### Data Collection Process and Data Items

Data were independently extracted by 2 authors (BS and SH) into a preprepared excel sheet. The following items were extracted: means and SDs at postintervention for pain interference, psychological distress and HRQoL, sample size, measures, mean age, percentage of females, diagnosis, mean years of pain, method of recruitment, and presence of contact with researchers or therapists. If no SDs were reported, they were calculated from the available SEs or CIs.

### Risk of Bias

In line with previous research, risk of bias within individual studies was assessed using the Cochrane Risk of Bias tool. Please see the published protocol for additional details [1]. Funnel plots and Egger tests were conducted to investigate publication bias across studies.

### Geometry of the Network

The network includes a node for each eHealth modality. In addition, the network contains both a control node (comprised wait list control and treatment-as-usual control groups) and an enhanced control node (eg, educational booklet).

### Summary Measures

Standardized mean differences (SMDs) between groups at postintervention and measures of uncertainty are reported. Additional summary measures such as treatment rankings and the probability of each modality arm being the best are reported.

### Planned Methods of Analysis

Random-effects pairwise meta-analyses of each available comparison were run as an exploratory analysis using Stata 13 (StataCorp LLC). These analyses were carried out on both the primary and secondary outcomes: pain interference, pain severity, psychological distress, and HRQoL.

An NMA random-effects model of the eHealth modalities used to deliver chronic pain interventions with the purpose of reducing pain interference was developed in WinBUGS 14 (MRC and Imperial College of Science, Technology and Medicine). This model was based on a Bayesian framework but was created with vague priors. The NMA returned pairwise comparisons between all modalities, rankings of the modalities and assessed the probability that each modality is the best. Tests of design inconsistency [53] and loop inconsistency [54] were run using Stata 13. Node splitting was conducted on comparisons with both direct and indirect evidence [55]. Additional information is provided in the protocol [1].

### Additional Analyses

As outlined in the protocol [1], the purpose of adding study-level covariates was to reduce heterogeneity by allowing the NMA to take account of additional information and minimize the differences between the studies within each modality. Covariates would be added to the model based on a reduction in the deviance information criterion (DIC). In this network, the added covariates did not have a significant effect. Sensitivity analyses investigating the influence of priors, initial values, length of burn-in, and testing convergence were carried out.

## Results

### Study Selection

**Figure 1:**
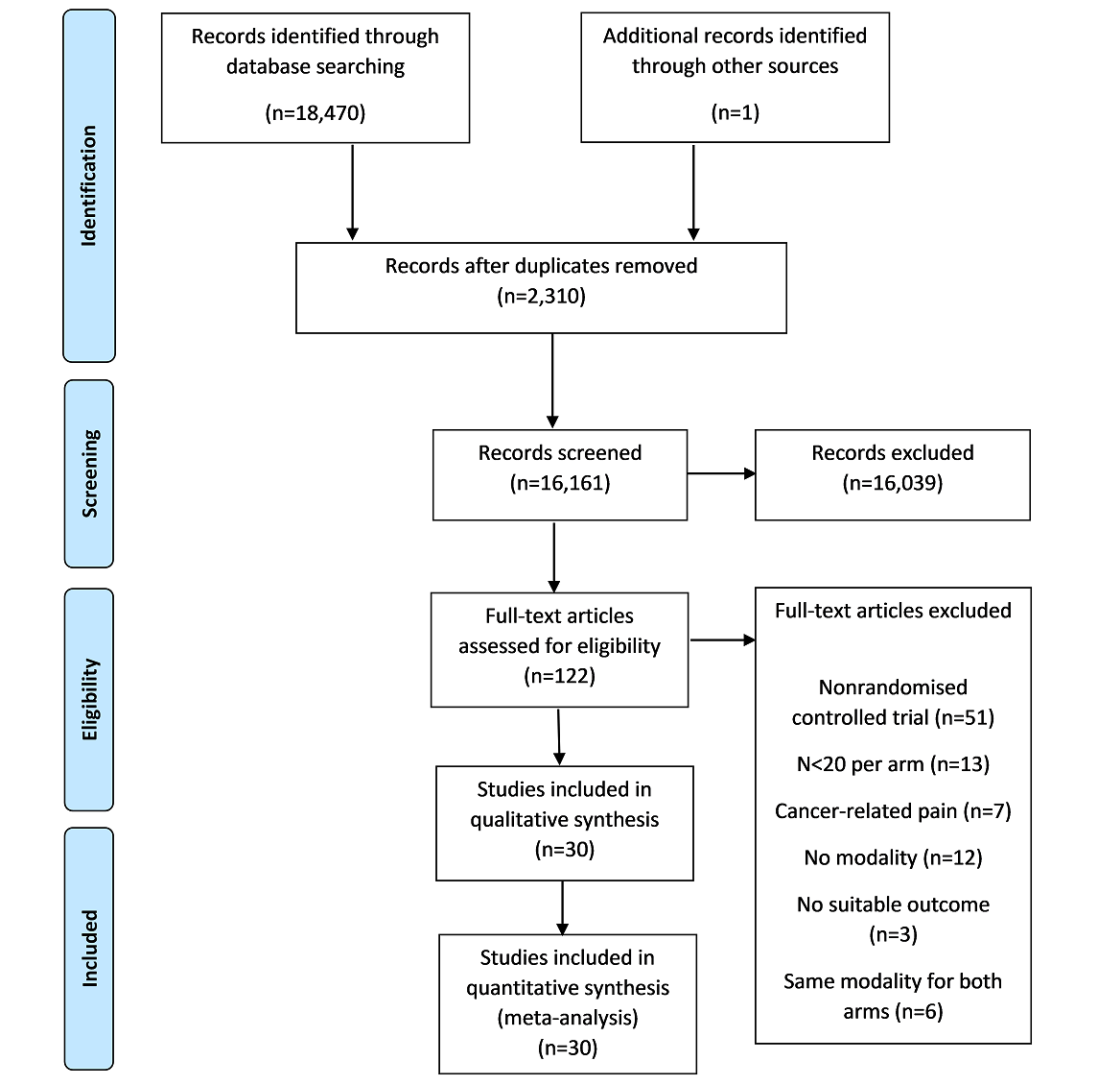
Flow diagram of studies assessed for eligibility.

The search returned 18,470 studies (Figure 1): PsycINFO (n=1913), MEDLINE (n=5286), EMBASE (n=10,479), and CENTRAL (n=792). There were 2310 studies that were excluded as duplicates and 16,039 studies excluded on the basis of title and abstract. In total, 122 potentially eligible studies were identified and then assessed on the basis of full text. Of these, 92 studies were excluded: 51 studies were not an RCT; 13 studies had less than 20 participants per arm at each time point; 7 studies had patients with cancer-related chronic pain; 12 studies did not deliver the intervention via an eHealth modality; 3 studies did not measure an appropriate pain outcome; and 6 studies consisted of 2 arms within the same node without an additional comparator. There were 30 studies that were included in the analysis.

### Study Characteristics

30 studies were included in this review. Each study arm was classified by the primary delivery method (eg, internet). Although the majority of intervention arms were compared with control arms, 1 study involved the comparison of 2 active treatments [56]. The 30 studies encompassed 61 arms: 23 internet-delivered arms [5-9,15,34,36,37,46,57-69]; 2 telephone [35,70]; 1 mobile app [71]; 2 virtual reality [14,72]; 1 videoconferencing [15]; 1 interactive voice response [12]; 25 control; and 13 enhanced controls (Table 2).

**Table 2:** Studies included in the review.

Study	Comparison	N^a^	Pain conditions / location	Average age (years)	Gender, female, n (%)	Attrition, n (%)
Berman (2009)	Internet (mind-body) versus control (WLC^b^)	89 (52, 37)	Nonspecific chronic pain	65.8	68 (87.2)	11 (12.4)
de Boer (2014)	Internet (CBT^c^) versus enhanced control (face-to-face CBT)	63 (33, 30)	Nonspecific chronic pain	52.1	32 (64)	13 (20.6)
Bromberg (2012)	Internet versus control (TAU^d^)	185 (92, 93)	Migraine or headache	42.6	165 (89)	19 (10.2)
Buhrman (2004)	Internet (CBT) versus control (WLC)	56 (22, 29)	Chronic back pain	44.6	35 (62.5)	5 (8.9)
Buhrman (2011)	Internet (CBT) versus control (WLC)	54 (26, 28)	Chronic back pain	43.2	37 (68.5)	4 (7.4%)
Carpenter (2012)	Internet (CBT) versus control (WLC)	141 (70, 71)	Chronic lower back pain	42.5	117 (83)	23 (16.3)
Chiauzzi (2010)	Internet (self-management) versus enhanced control (text-based material)	199 (95, 104)	Chronic back pain	46.1	134 (67.7)	15 (7.5)
Dear (2013)	Internet (CBT) versus control (WLC)	62 (31, 31)	Multiple pain conditions/sites	49	53 (85)	2 (3.2)
Dear (2015)	Internet (CBT) versus control (WLC)	472 (397, 75)	Multiple pain conditions/sites	50	375 (80)	50 (10.6)
Dear (2017)	Internet (CBT) versus enhanced control (workbook)	164 (76, 88)	Multiple pain conditions/sites	47.8	135 (82)	14 (8.5)
Devineni (2005)	Internet versus control (WLC)	86 (39, 47)	Migraine or headache	41.3	111 (79.6)	53 (38.1)
Garcia-Palacios (2015)	Virtual reality (activity management) versus control (TAU)	61 (31, 30)	Fibromyalgia syndrome	50.5	61 (100)	2 (3)
Herbert (2017)	Videoconferencing (ACT^e^) versus enhanced control (face-to-face ACT)	129 (65, 64)	Multiple pain conditions/sites	52	23 (17.8)	28 (21.7)
Kleiboer (2014)	Internet versus control (WLC)	368 (195, 173)	Migraine or headache	43.6	314 (85)	96 (26)
Krein (2013)	Internet (pedometer) versus enhanced control (pedometer)	229 (111, 118)	Chronic low back pain	51.6	29 (12.7)	22 (9.6)
Kristjánsdóttir (2013)	Mobile app (CBT) versus internet (CBT)	140 (70, 70)	Chronic widespread pain	44.2	140 (100)	40 (28.6)
Kroenke (2014)	Telephone (care management) versus control (TAU)	250 (124, 126)	Chronic musculoskeletal and chronic generalised pain	55.2	43 (17.2)	12 (4.8)
Leveille (2009)	Internet (health coaching) versus enhanced control (general health information)	241 (121, 120)	Chronic musculoskeletal pain	52.4	138 (57.3)	99 (41.1)
Lin (2017)	Internet (ACT) versus control (WLC)	302 (201, 101)	Multiple pain conditions/sites	51.7	254 (84.1)	73 (24.2)
Lorig (2008)	Internet (pain management) versus control (TAU)	855 (433, 422)	Arthritis or fibromyalgia	52.4	780 (91.2)	214 (25)
McBeth (2012)	Telephone (CBT) versus enhanced control (exercise) versus control (TAU)	442 (224, 109, 109)	Chronic widespread pain	56.2	307 (69.5)	81 (18.3)
Müller (2016)	Internet (positive psychology) versus control (text-based materials)	96 (51, 45)	Multiple pain conditions/sites	59.4	67 (69.8)	19 (19.8)
Naylor (2008)	Interactive voice response (CBT) versus control (TAU)	51 (26, 25)	Chronic musculoskeletal pain	46	44 (86)	0 (0)
Peters (2017)	Internet (positive psychology) versus control (WLC)	284 (233, 51)	Multiple pain conditions/sites	48.9	234 (84.7)	70 (24.6)
Ruehlman (2012)	Internet (CBT) versus control (WLC)	305 (162, 143)	Multiple pain conditions/sites	44.9	195(64)	64 (20.9)
Ström (2000)	Internet (applied relaxation) versus control (WLC)	102 (20, 25)	Headache related pain	36.7	69 (67.6)	57 (56)
Trompetter (2015)	Internet (ACT) versus control (WLC)	238 (161, 77)	Multiple pain conditions/sites	52.8	181 (76)	66 (27.7)
Williams (2010)	Internet (self-management) versus control (TAU)	118 (59, 59)	Fibromyalgia	50.5	112 (95)	12 (10.2)
Wilson (2015)	Internet (pain management) versus control (WLC)	114 (57, 57)	Chronic noncancer pain	49.3	72 (78)	34 (29.8)
Yilmaz Yelvar (2017)	Virtual reality (physiotherapy) versus enhanced control (physiotherapy)	46 (23, 23)	Non-specific low-back pain	49.6	28 (63.63)	2 (4.3)

^a^Total N randomized (Arm 1 N, Arm 2 N, Arm 3 N [where applicable]).

^b^WLC: waitlist control

^c^CBT: cognitive behavioral therapy

^d^TAU: treatment-as-usual.

^e^ACT: acceptance and commitment therapy.

A total of 5288 participants were included in the review. There were 3005 participants randomized to interventions delivered via an eHealth modality: internet (n=2509); telephone (n=305); videoconferencing (n=65); virtual reality (n=53); mobile apps (n=47); and interactive voice response (n=26).

### Risk of Bias Within Studies

The risk of bias summary is presented in Table 3. In total, 18 studies were considered to have been effectively randomized, 11 studies did not provide adequate information, and 1 study did not describe randomization and was judged to be at a high risk of bias. Furthermore, 10 studies used appropriate methods of allocation concealment, 18 studies did not appropriately describe their allocation methods, and 2 studies were judged as high risk, given that allocation was not blinded from research assistants. A total of 23 studies were not at risk of detection bias; the majority of these administered their assessments online. Furthermore, 7 studies were considered unclear. Although 15 studies provided clear information on their levels of attrition, 14 of them were judged to be unclear, with many failing to report differences between completers and noncompleters, and 1 study was considered to be at high risk of bias because of statistical differences between the completers and noncompleters. In total, 28 studies reported all outcomes and were free from selective reporting bias. In addition, 2 studies were judged to be of high risk because data could not be extracted. No other sources of bias were found for the 30 studies.

**Table 3:** Assessment of within-study bias.

Study	Adequate sequence generation	Allocation concealment	Blinding	Incomplete outcome data addressed	Free of selective reporting	Free of other bias
Berman (2009)	+^a^	−^b^	+	+	+	+
de Boer (2014)	+	+	+	+	+	+
Bromberg (2012)	+	?^c^	+	+	+	+
Buhrman (2004)	+	?	+	+	+	+
Buhrman (2011)	?	+	+	+	+	+
Carpenter (2012)	+	?	+	?	−	+
Chiauzzi (2010)	?	?	?	?	+	+
Dear (2013)	−	−	+	?	+	+
Dear (2015)	+	+	+	?	+	+
Dear (2017)	+	+	+	?	+	+
Devineni (2005)	+	?	?	+	+	+
Garcia-Palacios (2015)	+	?	?	?	+	+
Herbert (2017)	?	+	+	+	+	+
Kleiboer (2014)	+	+	+	+	+	+
Krein (2013)	+	?	?	?	+	+
Kristjánsdóttir (2013)	+	?	?	+	+	+
Kroenke (2014)	+	+	+	?	+	+
Leveille (2009)	?	?	+	+	+	+
Lin (2017)	+	?	+	?	+	+
Lorig (2008)	?	?	+	−	+	+
McBeth (2012)	+	+	+	+	+	+
Muller (2016)	+	?	+	?	+	+
Naylor (2008)	?	?	+	?	+	+
Peters (2017)	?	?	+	+	+	+
Ruehlman (2012)	?	?	+	+	+	+
Strom (2000)	?	?	?	?	−	+
Trompetter (2015)	+	?	+	+	+	+
Williams (2010)	+	+	+	?	+	+
Wilson (2015)	?	?	+	+	+	+
Yelvar (2017)	?	+	?	?	+	+

^a^The study satisfied the criteria.

^b^The study did not satisfy the criteria.

^c^Researchers were unable to determine if criteria were satisfied.

### Results of Individual Studies

The included studies indicate positive effects for interventions delivered via eHealth modalities in comparison with a control/enhanced control; 80% (24/30) of studies returned a reduction in pain interference, 69% (18/26) of studies returned a reduction in pain severity, 79% (19/24) of studies showed a decrease in psychological distress, and 67% (8/12) studies indicated an improvement in HRQoL.

Exploratory analyses were carried out on the primary outcome, pain interference, secondary outcomes, pain severity, psychological distress, and HRQoL. An NMA was conducted for the primary outcome, pain interference.

#### Exploratory Analysis

Exploratory pairwise meta-analyses were conducted where possible (Table 4).

**Table 4:** Exploratory analyses.

Comparison and outcome		Number of studies	Standardized mean difference	*P* value
**Internet** **versus** **control**				
	Pain interference	18	0.28	<.001
	Pain severity	15	0.2	<.001
	Psychological distress	16	0.35	.001
	Health-related quality of life	6	0.02	.80
**Internet** **versus** **enhanced control**				
	Pain interference	5	0.17	.55
	Pain severity	5	0.16	.57
	Psychological distress	4	0.14	.33
	Health-related quality of life	1	0.34	.26

##### Pain Interference

Data were extracted for pain interference, disability, functional interference, physical impairment, physical functioning, and headache disability, using a variety of measures: the Brief Pain Inventory (BPI), Visual Analogue Scale (VAS), Multidimensional Pain Inventory (MPI), Pain Disability Index, Survey of Pain Attitudes, Roland-Morris Disability Questionnaire, 36-Item Short-Form Health Survey (SF-36), Fibromyalgia Impact Questionnaire, Health Assessment Questionnaire–Disability Index, Profile of Chronic Pain-Screen, Headache Disability Index, Oswestry Disability Index, and the Migraine Disability Assessment. Pairwise meta-analyses indicate that internet-delivered interventions result in a small statistically significant reduction in pain interference when compared with a control group (*P*<.001).

##### Pain Severity

Of the included studies, 26 included a measure of pain severity. Data were extracted for pain severity, pain intensity, average pain, typical pain, activity pain, and pain severity using the following measures: BPI, VAS, MPI, PCP-S, Pain Assessment Questionnaire, Brief Pain Questionnaire, Numeric Rating Scale, Visual Numeric Scale, McGill Pain Questionnaire, headache or pain diaries, and study-specific measures [6,67]. Internet-delivered studies returned a small statistically significant reduction in pain severity when compared with a control group (*P*<.001). A second NMA was conducted on the basis of the effectiveness of eHealth modalities in reducing pain severity; a network map and results are provided in Multimedia Appendix 3.

##### Psychological Distress

Of the included studies, 24 used a measure of psychological distress. Data were extracted for depression, anxiety, mental health, and negative mood regulation using a variety of measures: the Hospital Anxiety and Depression Scale, Montgomery-Asberg Depression Rating Scale–Self Rated, Beck Depression Inventory, Negative Affect Scale, Depression Anxiety Stress Scales, Patient Health Questionnaire 9-Item, Personal Health Questionnaire Depression Scale, Short Form (SF) 8 Health Survey, SF-36, and Centre for Epidemiologic Studies Depression Scale. Internet-delivered interventions returned a statistically significant SMD of 0.35 when compared with a control (*P*=.001). Internet-delivered interventions did not return a statistically significant reduction in psychological distress when compared with an enhanced control (*P*=.33).

##### Health-Related Quality of Life

Data were available on HRQoL for 12 studies. This was measured in a variety of ways, including the Quality of Life Interview, Patient Global Impression of Change, the Quality of Life Index, General Health Questionnaire 12-Item, 12-Item Short Form Survey, and SF-36. No statistically significant differences were found in HRQoL between the internet-delivered interventions and a control (*P*=.80).

### Presentation of Network Structure

**Figure 2:**
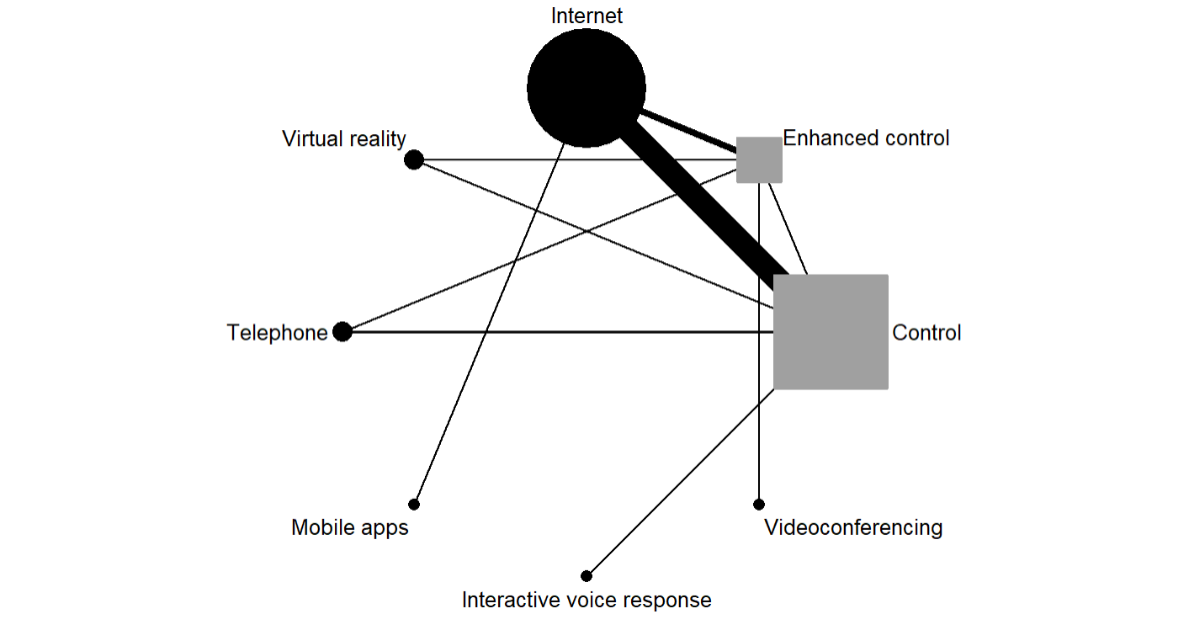
Network map of electronic health modalities for chronic pain.

The network map in Figure 2 demonstrates the available evidence for this reduction in pain interference network. For convenience, the circular nodes are eHealth modalities and the square nodes represent the control groups.

### Summary of Network Geometry

The available evidence was used to generate the network displayed in Figure 2. The number of studies behind each direct comparison is outlined in Table 5, which also includes the percentage of contribution that each comparison made to the entire network. As expected, the internet treatment versus control comparison contributes the highest percentage (17.67%) of evidence to the network. Some of the indirect comparisons required a long pathway to be generated (eg, comparing mobile apps with telephone-delivered interventions requires the direct evidence of the internet and control nodes). The comparisons based on longer paths were communicated with less precision [41].

**Table 5:** Contribution to the pain interference network.

Direct comparison	Studies, n	Contribution, %
Virtual reality versus control	1	8.18
Interactive voice response versus control	1	12.10
Internet versus control	18	17.67
Telephone versus control	2	13.23
Telephone versus enhanced control	1	9.41
Virtual reality versus enhanced control	1	5.60
Internet versus enhanced control	5	3.11
Videoconferencing versus enhanced control	1	10.94
Mobile apps versus internet	1	10.74
Enhanced control versus control	1	9.01

### Synthesis of Results

#### Network Meta-Analysis (Pain Interference)

A random-effects NMA based on the restricted maximum likelihood estimate was conducted to examine interventions delivered by eHealth modalities for the reduction of pain interference in chronic pain patients. The NMA suggests an SMD of 0.3, indicating a small difference between internet and the control (95% Credible Interval (CrI): 0.1 to 0.44) as expected by the exploratory analysis. In addition, an SMD of 0.28 was found between internet and the enhanced control (95% CrI: 0.002 to 0.55). The generated comparisons (Table 6) indicate that videoconferencing was significantly worse than all other modalities, bar interactive voice response.

The remaining comparisons had credible intervals containing 0, suggesting a high probability that the true comparison is not significant. Many of the credible intervals were very wide and expressed the uncertainty in the model’s estimates. It must be stressed that many of these comparisons were based on a low sample size and do not suggest that significance cannot be achieved with a greater number of studies.

Table 7 outlines the rankings of the modalities and the probability that they deliver the most effective interventions. The mobile apps and virtual reality arms were given the best ranking, at second (95% CrI 1 to 7; 95% CrI 1 to 6). Slightly less uncertainty surrounds the ranking of the internet arm, with a median value of 3 and a credible interval from 1 to 5. The videoconferencing arm had a ranking of 8.

Table 7 indicates that there is a 43% chance that mobile app–delivered interventions are the most effective at reducing pain interference. The available evidence suggests that there is a 0% chance that videoconferencing delivers the most effective interventions.

**Table 6:** Results of network meta-analysis (NMA): electronic health (eHealth) modalities delivering interventions for reducing pain interference. Data in italics are statistically significant.

Modality	Internet, SMD^a^ (CrI^b^)	Virtual reality, SMD (CrI)	Telephone, SMD (CrI)	Mobile apps, SMD (CrI)	Interactive voice response, SMD (CrI)	Videoconferencing, SMD (CrI)	Enhanced control, SMD (CrI)
Virtual reality	−0.16 (−0.77 to 0.44)	—^c^	—	—	—	—	—
Telephone	−0.01 (−0.57 to 0.55)	0.15 (−0.63 to 0.95)	—	—	—	—	—
Mobile apps	−0.21 (−0.95 to 0.54)	−0.04 (−1 to 0.92)	−0.19 (−1.12 to 0.74)	—	—	—	—
Interactive voice response	0.39 (−0.44 to 0.12)	0.55 (−0.46 to 1.57)	0.4 (−0.59 to 1.39)	0.6 (−0.52 to 1.72)	—	—	—
Videoconferencing	*1.59* *(0.82 to 2.36)*	*1.75 (0.81 to 2.69)*	*1.6 (0.67 to 2.54)*	*1.8 (0.72 to 2.87)*	*1.2 (0.06 to 2.33)*	—	—
Enhanced control	*0.28 (0.002 to 0.55)*	0.44 (−0.16 to 1.03)	0.29 (−0.29 to 0.87)	0.49 (−0.31 to 1.27)	−0.11 (−0.99 to 0.76)	*-1.32 (-2.1 to −0.53)*	—
Control	*0.3 (0.1 to 0.44)*	0.44 (−0.15 to 1.04)	0.29 (−0.26 to 0.83)	0.48 (−0.28 to 1.24)	−0.11 (−0.93 to 0.7)	− *1.31 (−2 to −0.59)*	−0.004 (−0.31 to 0.31)

^a^SMD: standardized mean difference.

^b^CrI: credible interval.

^c^Not applicable.

**Table 7:** Ranked effectiveness of modalities.

Modality	Median ranking^a^ (credible interval)	Probability^b^ (SD)
Internet	3 (1 to 5)	.04 (0.19)
Virtual reality	2 (1 to 6)	.34 (0.47)
Telephone	3 (1 to 7)	.15 (0.36)
Mobile apps	2 (1 to 7)	.43 (0.50)
Interactive voice response	7 (1 to 7)	.05 (0.21)
Videoconferencing	8 (8 to 8)	.000008 (0.003)
Enhanced control	6 (3 to 7)	.0007 (0.03)
Control	6 (4 to 7)	.00002 (0.004)

^a^Treatments ranked in order of comparative effectiveness.

^b^Probability of each treatment being the best (ie, most effective).

### Exploration for Inconsistency

Given that all of the studies included in this review were randomized, the assumption of transitivity is fulfilled. A test of loop inconsistency and a Lu-Ades test of design inconsistency revealed no evidence of inconsistency (*P*=.85 and *P*=.67, respectively). Node splitting returned no evidence of inconsistency when assessing differences between direct and indirect effects.

### Risk of Bias Across Studies

The funnel plot (see Multimedia Appendix 4) showed no indication of publication bias with the majority of studies falling within the bands. Most studies were clustered around the zero line and have relatively large SEs.

### Results of Additional Analyses

Sensitivity analyses were carried out to assess the fit of the model. A variety of different initial values were tested; the model was run with an extended burn-in of 200,000 iterations. Both a gamma and half normal prior were used to ensure that the normal prior was uninformative, and 500,000 and 700,000 iterations were run to ensure that 600,000 were adequate. In addition, the model was run with 2 chains, and history plots showed tight iterations, indicating no evidence of nonconvergence.

Additional covariates were added to the model to explore heterogeneity. The initial NMA model returned a DIC of 12.47. When the covariates were added to the model, the DIC did not significantly reduce and they were considered not to have added enough to the model to warrant inclusion (age [DIC=13.72], gender [DIC=12.79], length of intervention [DIC=12.84], attrition [DIC=13.15], measure [DIC=13.20], contact [DIC=13.03], analysis [DIC=12.99], and condition [DIC=12.99]).

## Discussion

### Principal Findings

The random-effects NMA returned pairwise comparisons between each of the eHealth modalities. The majority of these comparisons were not statistically significant; however, the network indicates that all eHealth modalities were significantly better than videoconferencing. On the basis of the currently available evidence, the network also promotes the use of internet to deliver interventions. This study created a ranked list of eHealth modalities used for chronic pain by conducting a systematic review with an NMA. Study findings tentatively indicated that mobile apps and virtual reality were the most effective eHealth modalities for delivering interventions for reducing pain interference. More specifically, the joint highest ranked modalities overall, according to NMA analyses, were mobile apps, with a 43% chance that this modality delivered the most effective intervention for reducing pain interference. Following this, virtual reality had a 34% chance and telephone had a 15% chance of being the most effective delivery method. Internet-delivered interventions have a 4% chance of being the most effective at reducing pain; however, there was more certainty regarding their positioning and effectiveness as they contributed the most papers [27] to the network (comparisons including internet contributed a total of 20.78% to the network). Although the analyses revealed important insights for the potential rank order of eHealth modalities for chronic pain interventions, only tentative conclusions regarding the most effective treatment types can be drawn, as there are limitations with this review.

### Strengths and Limitations

One limitation with this review is the disproportionate representation of different eHealth modalities included within the network. For example, of the 30 papers included in the analysis, internet was represented in 23 papers and telephone was represented in 2 papers, whereas, mobile apps, interactive voice response, and videoconferencing were each represented in only 1 paper. As a result, although we can be confident of the ranking of internet relative to the other modalities in the network, we cannot be confident of the rankings of the other modalities relative to internet. To explain further, if for example, an additional internet paper was added to this network, the modality rankings would not be anticipated to change, but if a new study based on another modality was added, then there is a chance that the modality rankings would change. However, this review is bound by the available evidence, and the current synthesis provides the first steps toward ranking which eHealth methodologies are more efficacious in the context of chronic pain.

It must also be noted that a contributing factor to the limited number of included papers and, therefore, eHealth modality types may have been the restrictive inclusion and exclusion criteria used in this study. For example, 51 studies were excluded for not being RCTs and 13 studies were excluded for not having 20 participants per arm for each time point. Therefore, had the eligibility criteria been more relaxed, arguably, more studies would have been included, allowing a larger network to be produced. However, the inclusion and exclusion criteria employed in this review followed on from a previous review in the area and the exacting criteria ensured that the included papers were of high quality and had low risk of bias [49].

Finally, because of the heterogeneous nature of intervention content, it may be contentious whether the current approach was optimal to identify the effectiveness of eHealth modalities relative to one another. For example, if each study in this review administered cognitive behavioral therapy (CBT) across each modality type, through accounting for differences in extracted variables (eg, age and gender), it could be reasonably assumed that any notable differences detected were because of the effect of the modality and not the intervention content (ie, CBT). However, although this was not the case in this network, it may also be debated that the aforementioned scenario would actually yield which eHealth modality is best for a particular treatment type (eg, CBT) and not which eHealth modality is most efficacious in the context of chronic pain. In any case, the scientific and clinical purposes of this review were to identify which eHealth modality, on the basis of the available evidence, delivers the most efficacious intervention for people living with chronic pain and not which intervention type (eg, CBT) works best with which eHealth modality.

Although there are certain limitations with this review, the findings provide support for previous research, yield tentative conclusions regarding the ranked efficacy of eHealth modalities in the context of chronic pain, and offer insight into further areas for investigation. Similar to previous research [49], the results from the exploratory meta-analysis highlight that internet-delivered interventions can reduce pain interference for people living with chronic pain. Interestingly, with regard to the results from the NMA, the 2 modalities found, albeit tentatively, to be most efficacious (virtual reality and mobile apps) are relatively new eHealth modalities compared with others in the review. The reason is not clear, but perhaps these modalities offer more immersive and convenient intervention pathways that appeal to participants.

Empowering individuals to take an active role in their own health care has been identified as a crucial factor for improving the quality of care and reducing health care costs [73-76]. This is particularly important for people with long-term health issues that require prolonged lifestyle modifications and adjustments [77-79]. Self-management of chronic/long-term illnesses through education and supportive interventions can not only decrease utilization of health care services but may also lead to improvements in clinical outcomes and overall quality of life [73]. Increasing patient engagement in health care interventions has thus become a priority for health care organizations, researchers, and policy makers. eHealth modalities offer tremendous potential to engage patients as they are flexible and can be tailored to individual patient’s needs, preferences, and circumstances [78]. However, as these technologies require actions that must be initiated and sustained by the individual, it is vital that these interventions are designed in an easily accessible and engaging manner. Perhaps as the research findings of this study tentatively show, interventions delivered via virtual reality and mobile apps could yield promising results in this area by virtue of their immersive and accessible design. In particular, research should focus on conducting interventions with mobile apps for chronic pain. With 93% of Irish consumers having access to a mobile phone [80], and a myriad of mobile apps targeting people with chronic pain (a recent review found 373 mobile apps for older adults with arthritic pain alone [81]), it is concerning that only 1 study included in this review delivered an intervention via a mobile app.

### Conclusions

In the wider context of eHealth, there are 2 areas for future work. The first would be to replicate the synthesis of this review with different chronic conditions. The second area for future work would be to create a core outcome set for eHealth interventions, a standardized set of eHealth intervention engagement outcomes measuring, for example, fidelity, participant engagement, and user experience. Often, a treatment can have an effect in person, but this effect may not transfer to an eHealth intervention. In such instances, it is quite possible that the eHealth execution and delivery was unsatisfactory and *not* that the intervention content cannot be adapted to an eHealth version. A core eHealth outcome set would assist in negating such issues.

In conclusion, from both a clinical and scientific perspective, previous research has outlined a need to compare eHealth modalities in the context of chronic pain. This research is the first to use a novel statistical method, namely, NMA, to quantitatively compare eHealth modalities in this context. Similar to previous research, the results suggest that internet interventions can improve pain interference, whereas more novel modalities (ie, mobile apps and virtual reality) are most likely to be effective, but more research on chronic pain eHealth is needed. Among many areas for future research, additional research examining underutilized eHealth modalities is recommended, and a core outcome set with regard to measuring engagement within eHealth interventions in general is paramount.

## Appendix

Multimedia Appendix 1 Detailed definitions/explanation of each electronic health modality.

Multimedia Appendix 2 Preferred Reporting Items for Systematic Reviews and Meta-analyses network meta-analysis checklist of items to include when reporting a systematic review involving a network meta-analysis.

Multimedia Appendix 3 Network meta-analysis of electronic health modalities used to deliver interventions for the reduction of pain severity in a chronic pain population.

Multimedia Appendix 4 Risk of bias across studies.

## Abbreviations

BPI: Brief Pain Inventory
CBT: cognitive behavioral therapy
CENTRAL: Cochrane Central Register of Controlled Trials
CrI: Credible Interval
DIC: deviance information criterion
eHealth: electronic health
EMBASE: Excerpta Medica dataBASE
HRQoL: health-related quality of life
MEDLINE: Medical Literature Analysis and Retrieval System Online
MPI: Multidimensional Pain Inventory
NMA: network meta-analysis
PRISMA: Preferred Reporting Items for Systematic Reviews and Meta-Analyses
RCT: randomized controlled trial
SMD: standardized mean difference
VAS: Visual Analogue Scale
