# Supplementary material for: An Evaluation of the Effectiveness of the Modalities Used to Deliver Electronic Health Interventions for Chronic Pain: Systematic Review With Network Meta-Analysis
Source: J Med Internet Res. 2019 Jul 17;21(7):e11086. doi: 10.2196/11086 (PMC6668295; doi:10.2196/11086)
Supplement: Multimedia Appendix 1 [file jmir_v21i7e11086_app1.pdf]

Appendix 1

Detailed Definitions/explanation of each eHealth modality:

Internet-based

Internet interventions are structured programmes that patients engage with by using computers or mobile devices. In addition to providing tailored educational information, they can "support self-management by facilitating goal setting, self-monitoring and providing behavioural/symptom-related feedback" (Geraghty et al., 2015, p.2).

Telephone supported

Telephone supported internet interventions usually involve a structured internet intervention, with the addition of telephone support from experienced practitioners with expertise in the content being delivered. The purpose of the telephone contact is generally to "provide support and encouragement for use of the internet intervention, and to address any concerns in relation to the internet-based content" (Geraghty et al., 2015).

Interactive voice response

Interactive Voice Response (IVR) technology is a "method for interaction between an individual and a computer through the medium of a telephone using the touch-tone keypad. Typically an automated script poses questions and the caller keys in responses using the telephone keypad" (Naylor et al., 2008, p.39).

Virtual reality

Virtual Reality (VR) is a three-dimensional computer generated environment, which allows the individual to explore, interact with, and manipulate objects by stimulating human senses (Riva, 2004).

Video teleconferencing

Video teleconferencing uses a combination of high-quality video and audio over Internet Protocol networks to facilitate real-time interactions between individuals and can be used for the management of health care, clinical support, and diagnostic purposes.

Mobile phone applications

Mobile phone health applications are mobile-based or mobile-enhanced programmes that deliver health-related services for smartphones, tablets, and other communication devices.

References:

Bennett G.G & Glasgow R.E. (2009). The delivery of public health interventions via the Internet: Actualizing their potential. Annual Review of Public Health, 30, 273–292.

Geraghty, A. W., Stanford, R., Little, P., Roberts, L., Foster, N. E., Hill, J. C., Hay, E., Stuart, B., Turner, D., … Yardley, L. (2015). Using an internet intervention to support self-management of low back pain in primary care: protocol for a randomised controlled feasibility trial (SupportBack). BMJ open, 5(9), e009524. doi:10.1136/bmjopen-2015-009524

Heapy, A., Sellinger, J., Higgins, D., Chatkoff, D., Bennett, T.C. & Kerns, R.D. (2007). Using Interactive Voice Response to Measure Pain and Quality of Life, Pain Medicine, 8(3): S145–S154 https://doi.org/10.1111/j.1526-4637.2007.00378.x

Riva, G. (2004). From Telehealth to E-Health: Internet and Distributed Virtual Reality in Health Care. CyberPsychology & Behavior, 3 (6). DOI: 10.1089/109493100452255
