# Supplementary material for: An Evaluation of the Effectiveness of the Modalities Used to Deliver Electronic Health Interventions for Chronic Pain: Systematic Review With Network Meta-Analysis
Source: J Med Internet Res. 2019 Jul 17;21(7):e11086. doi: 10.2196/11086 (PMC6668295; doi:10.2196/11086)
Supplement: Multimedia Appendix 3 [file jmir_v21i7e11086_app3.pdf]

Appendix 3

Network meta-analysis of eHealth modalities used to deliver interventions for the reduction of pain severity in a chronic pain population.

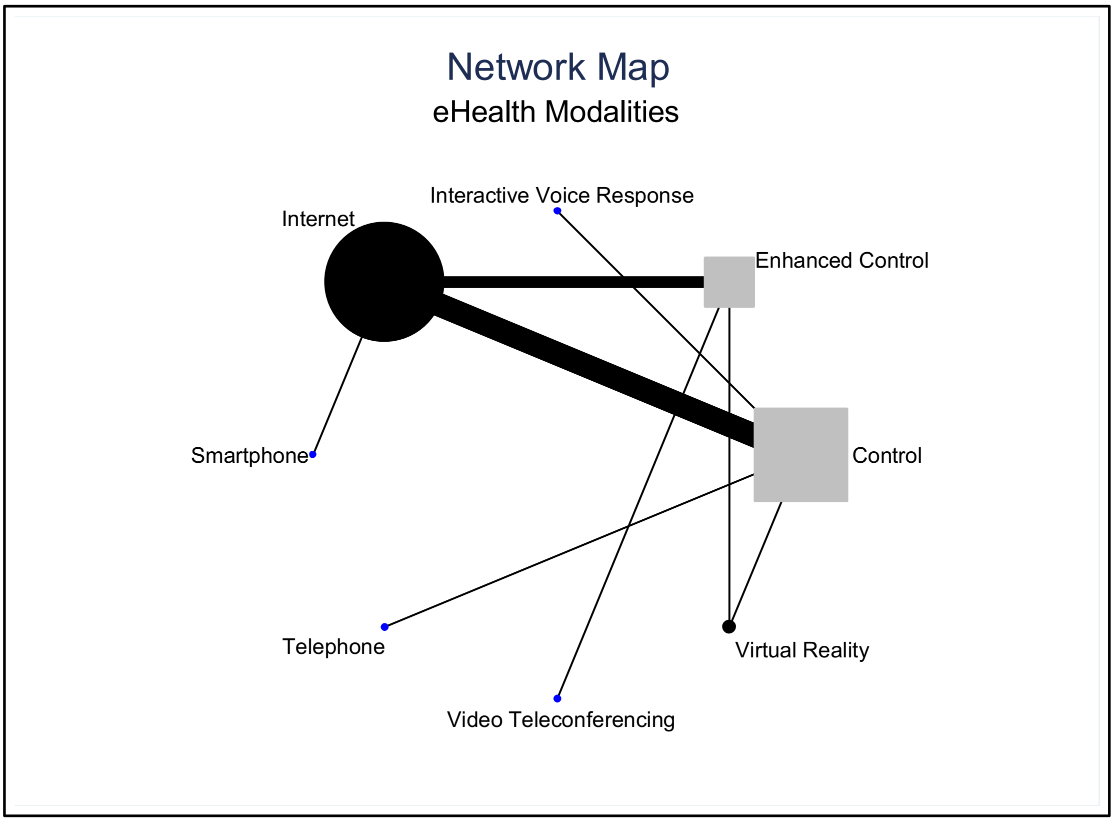

Pairwise comparisons between eHealth modalities delivering interventions for a reduction in pain severity

Modality	A	B	C	D	E	F	G
**B**	-.007 [-.67, .65]						
**C**	-.26 [-1.04, .51]	-.26 [-1.24, .73]					
**D**	.15 [-.67, .96]	.16 [-.88, 1.2]	.41 [-.71, 1.53]				
**E**	-.06 [-.97, .85]	-.05 [-1.15, 1.05]	.2 [-.95, 1.36]	-.21 [-1.43, 1.02]			
**F**	**2.22 [1.35, 3.08]**	**2.22 [1.18, 3.27]**	**2.48 [1.33, 3.63]**	**2.07 [.88, 3.26]**	**2.28 [1.02, 3.54]**		
**G**	**.309 [.02, .6]**	0.32 [-.33, .98]	.57 [-.25, 1.4]	.16 [-.7, 1.03]	0.37 [-.58, 1.33]	**-1.91 [-2.72, -1.1]**	

**H**	**.2** [**-.003, .41]**	.21 [-.44, .87]	.0.47 [-.27, 1.21]	.05 [-.79, .9]	.27 [-.62, 1.15]	**-2.01** **[-2.9, -1.13]**	-.11 [-.46, .25]

Data displayed as SMD [Credible Interval]

Bolded entries are statistically significant

A=Internet, B=Virtual reality, C=Telephone, D=Mobile applications, E=Interactive voice response, F=Video teleconferencing, G=Enhanced control, H=Control
