# Supplementary figures and images for: An Evaluation of the Effectiveness of the Modalities Used to Deliver Electronic Health Interventions for Chronic Pain: Systematic Review With Network Meta-Analysis
Source: J Med Internet Res. 2019 Jul 17;21(7):e11086. doi: 10.2196/11086 (PMC6668295; doi:10.2196/11086)

#### Appendix 4: Risk of bias across studies

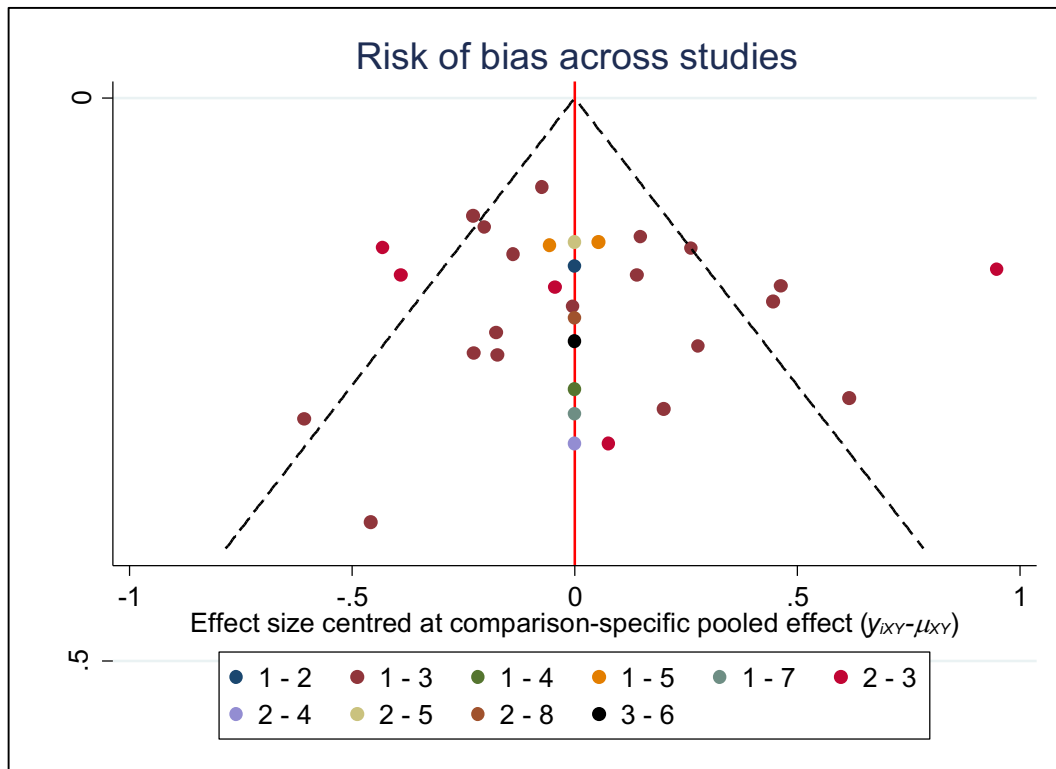

Supplement: Multimedia Appendix 4 [file jmir_v21i7e11086_app4.pdf]
